# Melanoma Development and Progression Are Associated with Rad6 Upregulation and **β**-Catenin Relocation to the Cell Membrane

**DOI:** 10.1155/2014/439205

**Published:** 2014-05-06

**Authors:** Karli Rosner, Darius R. Mehregan, Evangelia Kirou, Judith Abrams, Seongho Kim, Michelle Campbell, Jillian Frieder, Kelsey Lawrence, Brittany Haynes, Malathy P. V. Shekhar

**Affiliations:** ^1^Laboratory for Molecular Dermatology, Barbara Ann Karmanos Cancer Institute, 110 East Warren Avenue, Detroit, MI 48201, USA; ^2^Department of Dermatology, School of Medicine, Wayne State University, Detroit, MI 48201, USA; ^3^Center for Molecular Medicine and Genetics, School of Medicine, Wayne State University, Detroit, MI 48201, USA; ^4^Pinkus Dermatopathology Laboratory, Monroe, MI 48162, USA; ^5^Biostatistics, Barbara Ann Karmanos Cancer Institute, Detroit, MI 48201, USA; ^6^Department of Oncology, School of Medicine, Wayne State University, Detroit, MI 48201, USA; ^7^Department of Pathology, School of Medicine, Wayne State University, Detroit, MI 48201, USA; ^8^Barbara Ann Karmanos Cancer Institute, Detroit, MI 48201, USA

## Abstract

We have previously demonstrated that Rad6 and **β**-catenin enhance each other's expression through a positive feedback loop to promote breast cancer development/progression. While **β**-catenin has been implicated in melanoma pathogenesis, Rad6 function has not been investigated. Here, we examined the relationship between Rad6 and **β**-catenin in melanoma development and progression. Eighty-eight cutaneous tumors, 30 nevi, 29 primary melanoma, and 29 metastatic melanomas, were immunostained with anti-**β**-catenin and anti-Rad6 antibodies. Strong expression of Rad6 was observed in only 27% of nevi as compared to 100% of primary and 96% of metastatic melanomas. **β**-Catenin was strongly expressed in 97% of primary and 93% of metastatic melanomas, and unlike Rad6, in 93% of nevi. None of the tumors expressed nuclear **β**-catenin. **β**-Catenin was exclusively localized on the cell membrane of 55% of primary, 62% of metastatic melanomas, and only 10% of nevi. Cytoplasmic **β**-catenin was detected in 90% of nevi, 17% of primary, and 8% of metastatic melanoma, whereas 28% of primary and 30% of metastatic melanomas exhibited **β**-catenin at both locations. These data suggest that melanoma development and progression are associated with Rad6 upregulation and membranous redistribution of **β**-catenin and that **β**-catenin and Rad6 play independent roles in melanoma development.

## 1. Introduction

The Wnt/*β*-catenin pathway has been implicated in the development and progression of melanoma and a wide range of cancer types, including colorectal cancer, breast cancer, esophageal carcinoma, and liver cancer [1–3]. Under normal conditions, intracellular *β*-catenin levels are kept low through a multiprotein system that mediates *β*-catenin degradation [4]. Increases in expression and binding of certain Wnt ligands to Frizzled receptor or mutations in specific components of the *β*-catenin degradation assembly deactivate this regulatory mechanism. Consequently, *β*-catenin accumulates in the cytoplasm and translocates to the nucleus. Nuclear *β*-catenin stimulates transcription of a large number of TCF/*β*-catenin responsive genes that include cyclin D1, c-myc [5, 6], and the melanocyte-specific gene, microphthalmia-associated transcription factor MITF-M [7]. Thus, accumulation of nuclear *β*-catenin as observed in several cancer types is considered a marker of canonical Wnt/*β*-catenin pathway deregulation and unfavorable prognosis [3, 8].

Previous studies have reported an association between nuclear *β*-catenin accumulation and melanoma progression and suggested nuclear *β*-catenin to be a marker of poor prognosis [1, 7]. However, recent studies have shown that contrary to breast and colon cancer, metastatic progression of melanoma is associated with decreases in nuclear and cytoplasmic *β*-catenin expression [9, 10]. Moreover, clinical, genetic, and histological studies suggest that nuclear and cytoplasmic *β*-catenin may be used as biomarkers of good prognosis in melanoma [11–14].

Recently, HHR6, a human homologue of the yeast Rad6 gene and a principal component of the postreplication DNA repair pathway, has been identified as an important regulator of canonical Wnt/*β*-catenin signaling [15, 16]. HHR6, referred hereafter as Rad6, stabilizes *β*-catenin by polyubiquitin modifications that render *β*-catenin resistant to 26S proteasomal degradation [16]. Furthermore, Rad6 is a transcriptional target of *β*-catenin [17], thus revealing a positive feedback loop between *β*-catenin-mediated activation of Rad6 gene expression and Rad6-induced *β*-catenin stabilization.

Rad6 expression is low in normal breast tissues; however, increases in Rad6 protein expression are detected in hyperplastic, ductal carcinoma* in situ* (DCIS) and invasive breast carcinomas [15]. We have previously demonstrated a role for Rad6 in breast cancer progression through its regulatory effect on the canonical Wnt/*β*-catenin pathway [18]. Since the decrease/loss of nuclear *β*-catenin [9, 10], rather than increases as in breast cancer, is linked to melanoma progression, it is not known whether Rad6 and *β*-catenin work in concert to promote melanoma pathogenesis. Furthermore, Rad6 expression in the skin has not been investigated, and there are no data on the role of Rad6 in the pathogenesis of benign (nevi) and malignant (melanoma) melanocytic lesions. It is important to address this gap in knowledge because of the unmet medical need for new effective antimelanoma therapies and because Rad6 and *β*-catenin have been identified as therapeutic targets [19, 20].

In this study, we examined Rad6 and *β*-catenin expressions in serial sections of nevi, primary, and metastatic melanomas to determine their potential roles in melanoma development and metastatic progression. Our data suggest that membranous relocation of *β*-catenin and upregulation of Rad6 are independent markers of melanoma development and progression. We also offer a hypothesis that explains the role of membranous *β*-catenin relocation and decreasing cytoplasmic *β*-catenin in melanoma development, a phenomenon that has been linked to unfavorable prognosis [9, 21, 22].

## 2. Materials and Methods

### 2.1. Patients and Specimens

Cases were retrieved from the files of the Pinkus Dermatopathology Laboratory (PDL), a private dermatopathology laboratory located in Monroe, MI. Preserved paraffin-embedded tissue specimens collected for each case were assigned an accession code that excluded patient identifier information. Nevus and primary melanoma cases were selected for study using random numbers generated by a uniform random number generator (Stata/MP 13.1). The study groups consisted of 30 cases of melanocytic nevi, 29 cases of primary cutaneous melanoma, and 29 cases of metastatic cutaneous melanoma. The melanoma and melanocytic nevus subtypes are listed in Table 1. The study includes all metastatic cutaneous melanoma samples that were archived between 2010 and 2012. The number of cases for each nevus and primary melanoma subtype was determined to reflect the lesion's relative representation in cases obtained at the PDL during the above period. Atypical nevi were diagnosed using criteria originally proposed by Clark and lesion architecture as reviewed by Roth et al. [23]. This study was approved by the Wayne State University Human Investigation Committee.

### 2.2. Antibodies

Primary antibodies used in the study are as follows: (i) anti-*β*-catenin (IS702) was purchased from Dako (Glostrup, Denmark) and used in an undiluted form; and (ii) anti-Rad6 (ab31917) was purchased from Abcam (Cambridge, MA) and used at a 1 : 500 dilution. In humans, the yeast homologous Rad6 gene is duplicated and the proteins encoded by the two genes HHR6A (or Rad6A) and HHR6B (Rad6B) from chromosomes Xq24-q25 and 5q23-q31, respectively, share 95% identical amino acid residues [24]. Neither ab31917, our own Rad6 antibody [15], nor any other commercially available anti-Rad6 antibody is currently able to distinguish between Rad6A and Rad6B proteins. Therefore, rather than referring as Rad6A or Rad6B, we refer to the protein detected by the antibody as Rad6.

### 2.3. Immunohistochemistry Staining

Immunohistochemistry was performed as previously described [25]. Briefly, five-micrometer sections were deparaffinized in xylene and rehydrated in graded ethanol. For antigen retrieval, sections were microwaved in citrate buffer pH 6.0 (BioGenex, San Ramon, CA, USA) for 12 min at 95°C and cooled for 30 min prior to immunostaining. Sections were incubated with 3% hydrogen peroxide for 15 min, followed by incubation with primary antibody for 60 min. An automated immunostainer (i6000; BioGenex) was utilized for subsequent incubation steps: sections were incubated in MultiLink biotinylated anti-IgG for 20 min, horseradish peroxidase conjugated secondary antibody for 20 min, followed by development with 3-amino-9-ethyl-carbazole for 10 min (BioGenex). Sections were then counterstained with hematoxylin. All incubation steps were performed at room temperature, and sections were washed with Tris-buffered saline between incubations.

### 2.4. Controls

Lung and colon cancer tissues were included as positive controls for immunostaining with anti-*β*-catenin antibody, and breast cancer tissues were included as positive controls for staining with anti-Rad6 antibody. Tissue sections incubated without primary antibody served as negative controls.

### 2.5. Cell Enumeration

Stained sections were independently enumerated by two coauthors (D. R. Mehregan and M. Campbell), who were blinded to patient medical records for each case. Blinded enumeration was performed under light microscopy at 400x magnification, and an ocular grid consisting of a simple square lattice of 100 test points was utilized to count the number of positively and negatively stained melanomas or nevus cells for each section. Per section, the total number of positively and negatively stained cells was counted for each of three sequential horizontal fields. The mean value of the three fields was used to estimate the relative density of cells in a specimen. To increase assessment accuracy, all positively and negatively stained melanomas or nevus cells in a visual field were individually counted as opposed to being graded as ranges of percentages. Enumeration data were reviewed independently by each evaluator. When independent readings for positively stained cells differed by 20% for a given section, both evaluators reviewed the section together to establish a consensus reading. A specimen was considered negative if less than 4% of the cells were immunostained for Rad6 or *β*-catenin. A tumor was considered to be stained with high intensity if >50% of the cells in a specimen expressed Rad6 or *β*-catenin, similar to the criteria used by Mineta et al. [26].

### 2.6. Statistical Analysis

Kruskal-Wallis tests were used to compare groups on the basis of continuous variables such as age and percent positive cells. Chi-square tests for differences in proportions were used to compare groups on the basis of categorical variables such as gender and *β*-catenin localization. Spearman's rank correlation was used to assess the pairwise association between age and percent of Rad6 positive and *β*-catenin positive cells. Multinomial logistic regression was used to assess the simultaneous association of Rad6 and age with diagnostic group. Adjustments were made for multiple comparisons using Wilcoxon rank sum tests with Bonferroni correction for pairwise comparisons.

## 3. Results

Our analysis included 30 individuals diagnosed with nevi, 29 with primary melanoma, and 29 with metastatic melanoma (Table 1). These groups differed marginally with respect to gender (*P* = 0.08) and significantly in age (*P* = 0.0001). Significant age differences were observed between individuals with nevi and those with either primary melanomas (*P* = 0.02) or metastatic melanomas (*P* = 0.0001). In contrast, no age difference is observed between individuals with primary and metastatic melanomas (*P* = 0.27). Significant differences in age were also observed between groups defined by *β*-catenin localization (*P* = 0.007). Individuals with *β*-catenin localized in the cytoplasm were significantly younger than individuals with *β*-catenin localization at the cell membrane (*P* = 0.02) and marginally younger than individuals with *β*-catenin localization at both the cytoplasm and the cell membrane (*P* = 0.05). When age was categorized as <50, 50–60, and >60 years, there were statistically significant differences in Rad6 expression between the groups (*P* = 0.0008), although there is substantial variability (Table 2). Median Rad6 is greater in the group of people older than 60 years compared to the 50–60 years old group (*P* = 0.04) and <50 years old group (*P* = 0.001). A 20% discrepancy of positively stained cells between the two evaluators was observed in fewer than 5% of the cases, and these cases were evaluated together to establish a consensus reading.

### 3.1. *β*-Catenin Immunostaining

**Figure 1:**
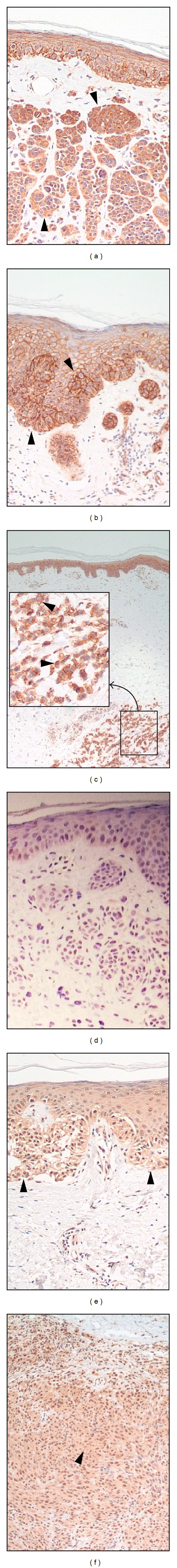
Representative pictures of *β*-catenin ((a)–(c)) and Rad6 ((d)–(f)) staining in nevus ((a), (d)), primary melanoma ((b), (e)), and metastatic melanoma ((c), (f)). Closed arrowheads point to positively immunostained cells. The highlighted square in panel (c) is magnified. Original magnification ×400.

**Figure 2:**
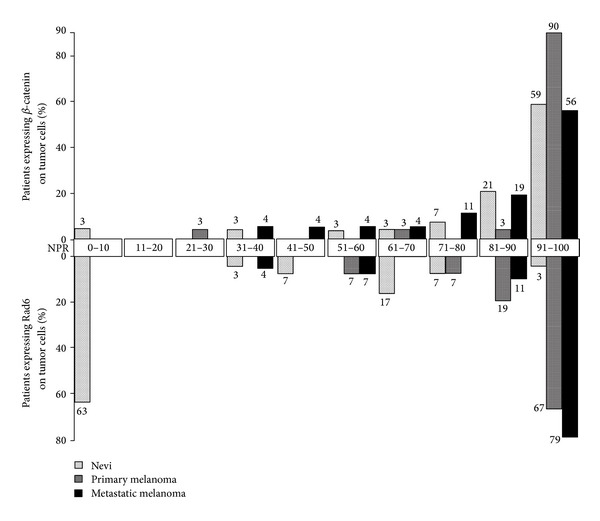
Percentages of nevi, primary melanomas, and metastatic melanomas with Rad6 and *β*-catenin positive cells shown in increments of 10 percentage points.

**Figure 3:**
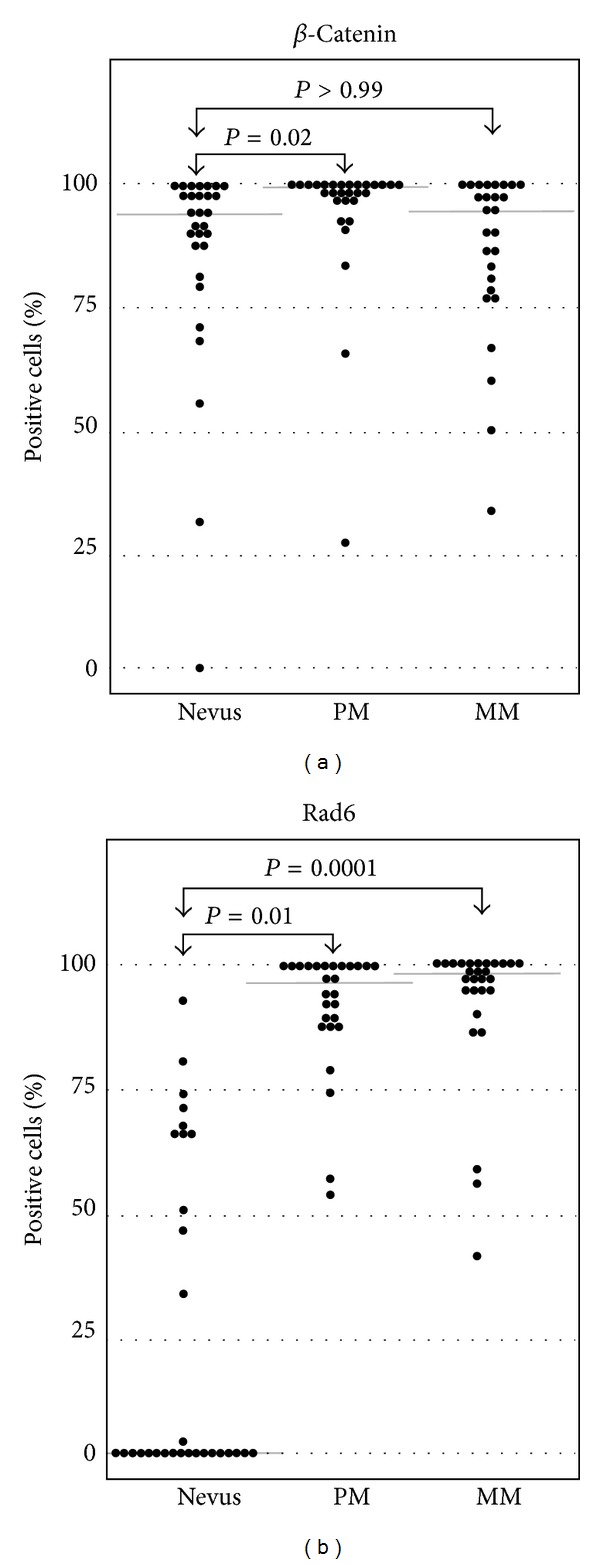
Boxplots of Rad6 and *β*-catenin positive cells in nevi, primary melanoma (PM), and metastatic melanoma (MM). Kruskal-Wallis tests showed that there are significantly more Rad6 positive cells in primary and metastatic melanomas as compared to nevi. Median values are indicated by gray horizontal lines.

Melanoma development and progression were not associated with significant changes in percentage of specimens expressing *β*-catenin. *β*-Catenin staining was observed in 97% of nevi and in all primary and metastatic melanomas. Also, the percentages of nevi (93%), primary melanoma (97%), and metastatic melanoma (93%) that expressed *β*-catenin in more than 50% of the cells did not differ significantly (Figures 1, 2, and 3). However, significant differences were observed between the percentages of nevi (59%), primary melanoma (90%), and metastatic melanoma (56%) that expressed *β*-catenin in more than 90% of the cells (*P* = 0.02; Figure 2). These differences were greatly impacted by the percentage of primary melanomas (48%) that expressed *β*-catenin in 100% of the cells, which was approximately twofold higher than the percentages of nevi (21%) or metastatic melanoma (26%) (data not shown).

### 3.2. Intracellular Localization of *β*-Catenin

**Figure 4:**
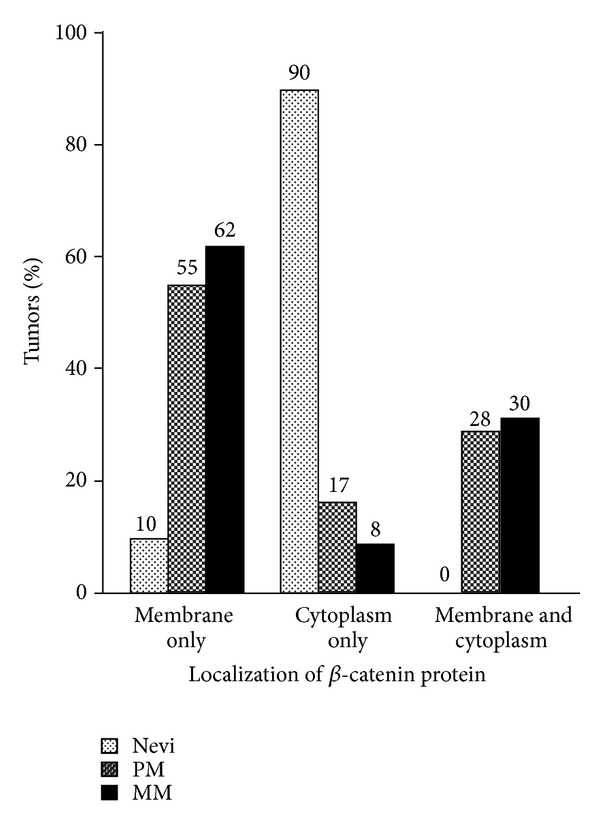
Subcellular localization of *β*-catenin in nevi, primary melanomas (PM), and metastatic melanomas (MM). Percentages of tumors expressing *β*-catenin exclusively in the cytoplasm or on cell membrane, or in both compartments.

None of the nevi or melanomas expressed *β*-catenin in the nucleus (Figure 1). The percentage of tumors that expressed membranous *β*-catenin increased dramatically from nevi (10%) to primary and metastatic melanomas (83% and 93%, resp.; *P* < 0.0001; Figure 4). Concurrently with this increase, the percentage of tumors that expressed cytoplasmic *β*-catenin decreased from nevi (90%) to primary and metastatic melanomas (45% and 38%, resp.; *P* < 0.0001; Figure 4). In contrast, no significant differences were observed between the percentages of primary and metastatic melanomas that expressed *β*-catenin at either the membrane (83% and 93%, resp.; *P* = 0.289) or the cytoplasm (45% and 38%, resp.; *P* = 0.633; Figure 4). While all four nevi types (junctional, intradermal, compound, and atypical) expressed *β*-catenin in the cytoplasm, only junctional and atypical nevi expressed *β*-catenin at the plasma membrane. As opposed to nevi types, the three primary melanoma types examined (superficial spreading, nodular, and lentigo maligna) did not differ in *β*-catenin localization as *β*-catenin was localized at the plasma membrane, in the cytoplasm, or in both (Figure 4).

### 3.3. Rad6 Immunostaining

The majority of nevi (63%) did not express Rad6. Conversely, all primary melanomas (100%) and the majority of metastatic melanomas (96%) exhibited greater than 50% Rad6 expression. The increase in tumor populations expressing Rad6 from 37% of nevi to 100% of primary and metastatic melanomas was significant (*P* = 0.0001; Figures 1, 2, and 3). Melanoma progression from primary to metastatic disease was not associated with changes in the (i) percentage of melanomas expressing Rad6 (100% of primary and metastatic melanomas), or (ii) percentage of melanomas expressing Rad6 in more than 50% of the tumor cells (100% and 96% of primary and metastatic melanomas, resp.). The increase in proportion of tumor populations, expressing Rad6 in more than 50% of the cells in primary melanoma (67%) versus metastatic melanoma (79%) was not significant (*P* = 0.37; Figure 2). This study was not designed to test whether the distribution of the tumor cells positive for Rad6 is the same between the subtypes of nevi. However, the percentages of benign tumors that lacked Rad6 were similar between atypical nevi (62%) and the group of other three nevi types (59%).

### 3.4. Rad6 as a Putative Biomarker for Differentiating Nevi from Melanoma

Interestingly, only one of the 30 nevi (atypical nevus, 3%) expressed Rad6 in >80% of the cells, and none of the primary and metastatic melanomas expressed Rad6 in <40% of the cells (Figure 2). These results prompted us to examine whether Rad6 expression can serve as a marker for histological diagnosis of melanoma. Using a multiple logistic regression model, we found that the strength of Rad6 expression is a strong predictor of melanoma (*P* < 0.001) even when age group (*P* = 0.65) and gender (*P* = 0.24) are included in the model. The model predicts that every 1% increase in Rad6 expression results in a 9% increase in the probability that a lesion is melanoma. If we assume that a predicted probability of >0.5 indicates melanoma, the model with only Rad6 has sensitivity of 93% and specificity of 80%. These results are very encouraging; however, they need to be validated in a larger study.

### 3.5. Correlation between *β*-Catenin and Rad6

The expression profiles of *β*-catenin and Rad6 differed considerably in nevi. Approximately 93% of nevi expressed *β*-catenin in more than 50% of the cells, whereas only 27% of the same population of nevi expressed Rad6 (Figure 2). *β*-Catenin and Rad6 expressions in nevi were not significantly correlated (*r* = 0.06; *P* = 0.77). There is a 2.7-fold difference in the percentage of primary melanomas (100%) expressing Rad6 compared to nevi (37%) and virtually no difference in *β*-catenin expression between primary and metastatic melanomas (100%). Accordingly, Rad6 and *β*-catenin expressions in primary melanoma were not correlated (*r* < 0.001, *P* > 0.99). A significant correlation between Rad6 and *β*-catenin positive cells was observed in metastatic melanoma (*r* = 0.45, *P* = 0.02). However, this association diminished (*r* = 0.40, *P* = 0.05) following the exclusion of two observations which are disproportionally influential (one with <50% positive Rad6 and one with <50% positive *β*-catenin).

## 4. Discussion

This is the first study to characterize Rad6 expression in cutaneous benign and malignant melanocytic tumors. In this study, we examined the association between Rad6 and *β*-catenin expressions in benign and malignant melanocytic tumors to determine whether Rad6 works in concert with *β*-catenin to influence melanoma development and progression. Rad6 and *β*-catenin positively regulate each other in breast cancer [15, 18]. However, while *β*-catenin has been implicated in the pathogenesis of melanoma and other cancer types, data about the role of Rad6 in cancer pathogenesis are mostly limited to breast cancer. Approximately 30% of melanomas develop in preexisting benign melanocytic neoplasms (nevi) [27]. Therefore, we hypothesized that comparison of Rad6 and *β*-catenin expressions in the same nevi and melanoma tumors would help determine whether these two signals collaborate to promote melanoma development and progression as they do in breast cancer [15, 28].

### 4.1. The Percentage of Samples Expressing *β*-Catenin Was Equally High in Nevi and Melanoma and Does Not Support a Central Role for *β*-Catenin Level in Melanoma Initiation and Progression

Accumulation of nuclear and cytoplasmic *β*-catenin has been implicated in driving the development and progression of several cancer types (e.g., colon and ovarian cancers) [29–31]. However, our results show that the expression levels of *β*-catenin do not contribute to melanoma initiation and progression since no difference in *β*-catenin levels was found between nevi, primary melanoma, and metastatic melanoma (93%–97% of all samples expressed *β*-catenin in >50% of the tumor cells). The high expression levels of *β*-catenin are in line with the crucial role of *β*-catenin in differentiation and proliferation of both normal melanocytes and metastatic melanoma cells [32]. Also, our findings are in agreement with previous reports of positive *β*-catenin staining in nevi (100%) and primary melanoma (95%, 94%) but are higher than reported in metastatic melanoma (75%, 68%) [9, 21]. The variation in expression of *β*-catenin levels in metastatic melanomas between the studies can be attributed to differences in the type of metastatic tissues. While we studied only melanoma metastases to the skin, other studies either obtained 58% of their specimens from lymph nodes, tonsil, and liver or did not identify the anatomical site of their metastases [9, 21]. Furthermore, different anatomical sites may regulate dissimilar antigen expressions in metastases that originate from the same primary tumor in the same patient [33, 34].

### 4.2. Nuclear *β*-Catenin Was Absent from All Nevi and Melanomas, Indicating Its Low Usefulness as a Prognostic Marker in Melanoma

Previous studies have shown higher percentages of nuclear *β*-catenin in nevi than in melanoma (84% versus 33%, and 44% versus 15%) [9, 22]. Those observations provided the basis for the currently held concept that loss of nuclear and cytoplasmic *β*-catenin suggest poor prognosis and decreased overall survival of melanoma patients [12, 22]. In light of these data, the absence of nuclear *β*-catenin in all the nevi and melanomas analyzed in our study was surprising. Usage of different anti-*β*-catenin antibodies may explain in part the discrepancy in nuclear *β*-catenin expression observed between the studies. However, our results are consistent with the lack of nuclear *β*-catenin reported in four studies which comprised 57 nevi, more than 55 primary melanomas, and 20 metastatic melanomas [21, 35–37]. Moreover, nuclear *β*-catenin was not found in either the nevus portion or the melanoma portion of 15 cutaneous lesions and was absent in additional 42 primary melanomas [38]. In another study of 70 primary melanomas, nuclear *β*-catenin was reported in only 6.4% of the melanomas [39]. Finally, in a study of 230 primary and metastatic melanomas, nuclear *β*-catenin was reported in only 13 cases (5.6%) and therefore those cases were excluded from analysis [21]. Taken together, the absence or negligible amount of nuclear *β*-catenin detected in the aforementioned studies as well as ours suggests possible extranuclear roles for *β*-catenin in nevi and melanoma. This notion is supported by a role for cytoplasmic *β*-catenin to execute functions that do not require nuclear translocation (e.g., activation of MAP kinase p38 and NF-kB) [37, 40].

### 4.3. Nevus to Melanoma Progression Is Associated with Cytoplasmic to Membranous Translocation of *β*-Catenin

A major finding of this study is the association between melanoma development and intracellular redistribution of *β*-catenin. The percentage of cases that expressed *β*-catenin on the cell membrane increased dramatically from 10% in nevi to 83% and 93% in primary and metastatic melanomas, respectively. Concurrently, the percentage of cases that expressed cytoplasmic *β*-catenin decreased from 90% in nevi to 45% and 39% in primary and metastatic melanomas, respectively (Figure 4). We hypothesize that the relocation of *β*-catenin from the cytoplasm to the cell membrane may serve as a deactivating mechanism of canonical Wnt/*β*-catenin signaling and that the resulting reduction in cytoplasmic *β*-catenin level may contribute to the malignant transformation of melanocytic nevi. The proposed hypothesis is supported by the following observations: (i) as in our study, Bachmann et al. also reported an association between nevus to melanoma development and relocation of *β*-catenin to the cell membrane [22]. Nevertheless, the authors of that study did not offer an explanation for their observation; (ii) our analysis of the data of Kagashita et al. showed *β*-catenin decrease in the cytoplasm and increase at the cell membrane and that these changes in *β*-catenin distribution corresponded with the malignant transition of nevi [9]; (iii) Wnt4 signal has been identified as a mechanism that can drive *β*-catenin relocation from cytoplasm to cell membrane [41]; and (iv) *β*-catenin relocation from cytoplasm to cell membrane has been reported to block *β*-catenin signaling in a human embryonic kidney (HEK293) cell line [41]. Of note, this hypothesis can explain how despite the abundant *β*-catenin expression in melanoma [1, 7], cytoplasmic *β*-catenin is selectively decreased, a phenomenon that has been associated with unfavorable melanoma prognosis [9, 21, 22]. Our current efforts are directed towards determining if the increases in membranous *β*-catenin observed in primary and metastatic melanomas result from relocation of existing molecules in the cytoplasm or deposition of newly generated *β*-catenin at the membranous site.

### 4.4. Rad6 Plays a Role in Melanoma Development and Progression, but Not in Nevi Formation

Rad6 has been implicated in early breast cancer development since an increase in Rad6 levels is observed in adenosis and benign hyperplasias as compared to normal tissue [15]. In contrast, our findings do not support Rad6 to play a similar role in nevus formation as in benign breast neoplasia, since 63% of the nevi were negative for Rad6. Rad6 has also been implicated in breast cancer progression because Rad6 levels increase with progression from ductal carcinoma* in situ* to invasive primary carcinoma and metastatic cancer [15, 28]. In accordance with the upregulation of Rad6 in early stages of breast cancer development as compared to benign hyperplasia [15, 17], we observed a striking increase in Rad6 expression in primary melanoma when compared to nevi. While all primary melanomas displayed strong Rad6 staining (>50% of the tumor cells), Rad6 was negative in 63% of the nevi. These findings suggest that Rad6 may play a role in malignant transformation of nevi as in breast cancer. Progression of melanoma from primary to metastatic disease was not significantly associated with further changes in the percentage of tumors expressing Rad6 or Rad expression intensity as >50% of tumor cells stained positively in 100% and 96% of primary melanomas and metastatic melanomas, respectively. These findings suggest that Rad6 may play a sustained role in melanoma metastasis as it does in melanoma development.

### 4.5. Correlation between *β*-Catenin and Rad6

In benign and malignant breast tumors, Rad6 stabilizes *β*-catenin, and, in turn, *β*-catenin positively upregulates Rad6 transcription [15–17]. However, this direct positive correlation between *β*-catenin and Rad6 expression does not appear to be conserved in melanoma as the expression profiles of *β*-catenin and Rad6 differed considerably in nevi. Approximately 93% of nevi expressed *β*-catenin compared to only 27% of nevi that expressed Rad6 in more than 50% of their cells (Figure 2). These observations suggest that the high *β*-catenin expression in nevi is likely driven by regulators other than Rad6. At first glance, it would appear that *β*-catenin and Rad6 expressions are correlated in primary melanoma because these proteins were coexpressed in approximately all primary melanomas. Also, these findings correspond to the 80% correlation between Rad6 and *β*-catenin expressions in primary breast cancer [17]. However, it is unlikely that the high Rad6 expression in primary melanoma is driven by the concurrent high *β*-catenin expression, because Rad6 expression is low in nevi despite the presence of high cytoplasmic *β*-catenin expression that is comparable to primary melanoma. This notion is confirmed by lack of statistical correlation between Rad6 and *β*-catenin expressions in primary melanoma. *β*-Catenin is not the only activator of Rad6; for instance, Rad6 is activated by nerve growth factor in nervous tissue [42]. Therefore, it is conceivable that, in primary melanoma, Rad6 expression is regulated by yet unidentified activators. We also demonstrated that further progression of melanoma from primary to metastatic disease is not associated with a correlation between the *β*-catenin and Rad6 expressions. Taken together, our study does not support a direct positive interaction between *β*-catenin and Rad6 in either benign or malignant melanocytic tumors.

## 5. Conclusion

We characterized for the first time Rad6 expression in cutaneous benign and malignant melanocytic tumors. We are showing a striking upregulation of Rad6 from a negative expression in most benign melanocytic tumors to 100% of primary and metastatic melanomas. These findings strongly suggest a role for Rad6 in the development of primary melanoma and metastatic disease. We show that in contrast to Rad6, *β*-catenin is expressed in more than 50% of the tumor cells in almost all nevi and melanoma tumors. Taken together, in contrast to the Rad6 *β*-catenin positive relationship in breast cancer [15–17], our study does not support a similar positive interaction between *β*-catenin and Rad6 in benign or malignant melanocytic tumors. Finally, our findings suggest a role for the cytoplasmic to membrane translocation of *β*-catenin in the development of primary melanoma. Future studies will determine whether newly generated *β*-catenin at the membranous site coincide with *β*-catenin translocation from the cytoplasm.

## Figures and Tables

**Table 1:** Association of melanocytic nevi and melanoma with demographic characteristics of the patients.

Melanocytic tumor type and subtype		No. Cases		Gender		Age
		*n*	Total *N*	M	F	Median (Range)
Nevi	Junctional nevus	5	30	11	19	43 (33–53)
	Intradermal nevus	7				
	Compound nevus	10				
	Atypical nevus	8				

PM	SSMM	17	29	14	15	58 (48–68)
	Nodular Melanoma	7				
	Lentigo Malignant Melanoma	5				

MM	Metastatic Melanoma	29	29	19	10	67 (58–76)

significance				*P* = 0.08		*P* = 0.00025

M: male; F: female; CI: confidence interval; PM: primary melanoma; MM: metastatic melanoma; SSM: superficial spreading melanoma.

**Table 2:** Rad6 positive cells by age groups.

Age (years)	*N*	Rad6 Percent Median (IQR)
<50	31	65 (0, 96)
50–60	16	86 (24, 95)
>60	38	96 (88, 100)

*N*: number of patients, IQR: interquartile range.

## Abbreviations

HHR6: Human homologue of the yeast Rad6
DCIS: Ductal carcinoma* in situ*
PDL: Pinkus Dermatopathology Laboratory.
